# Ficolin-1 and Ficolin-3 Plasma Levels Are Altered in HIV and HIV/HCV Coinfected Patients From Southern Brazil

**DOI:** 10.3389/fimmu.2018.02292

**Published:** 2018-10-08

**Authors:** Maria Regina Tizzot, Kárita Cláudia Freitas Lidani, Fabiana Antunes Andrade, Hellen Weinschutz Mendes, Marcia Holsbach Beltrame, Edna Reiche, Steffen Thiel, Jens C. Jensenius, Iara J. de Messias-Reason

**Affiliations:** ^1^Laboratory of Molecular Immunopathology, Department of Medical Pathology, Federal University of Paraná, Curitiba, Brazil; ^2^Clinic Hospital, Estate University of Londrina, Londrina, Brazil; ^3^Department of Biomedicine, Aarhus University, Aarhus, Denmark

**Keywords:** Ficolin-1, Ficolin-3, complement system, HIV infection, hepatitis C virus

## Abstract

The complement system is a key component of the innate immune system, participating in the surveillance against infectious agents. Once activated by one of the three different pathways, complement mediates cell lysis, opsonization, signalizes pathogens for phagocytosis and induces the adaptive immune response. The lectin pathway is constituted by several soluble and membrane bound proteins, called pattern recognition molecules (PRM), including mannose binding lectin (MBL), Ficolins-1, -2, and -3, and Collectin 11. These PRMs act on complement activation as recognition molecules of pathogen-associated molecular patterns (PAMPs) such as N-acetylated, found in glycoproteins of viral envelopes. In this study, Ficolin-1 and Ficolin-3 plasma levels were evaluated in 178 HIV patients (93 HIV; 85 HIV/HCV) and 85 controls from southern Brazil. Demographic and clinical-laboratory findings were obtained during medical interview and from medical records. All parameters were assessed by logistic regression, adjusted for age, ancestry, and sex. Significantly lower levels of Ficolin-1 were observed in HIV/HCV coinfected when compared to HIV patients (*p* = 0.005, median = 516 vs. 667 ng/ul, respectively) and to controls (*p* < 0.0001, 1186 ng/ul). Ficolin-1 levels were lower in males than in females among HIV patients (*p* = 0.03) and controls (*p* = 0.0003), but no association of Ficolin-1 levels with AIDS was observed. On the other hand, Ficolin-3 levels were significantly lower in controls when compared to HIV (*p* < 0.0001, medians 18,240 vs. 44,030 ng/ml, respectively) and HIV/HCV coinfected (*p* < 0.0001, 40,351 ng/ml) patients. There was no correlation between Ficolin-1 and Ficolin-3 levels and age, HIV viral load or opportunistic infections. However, Ficolin-3 showed a positive correlation with T CD4 cell counts in HIV monoinfected patients (*p* = 0.007). We provide here the first assessment of Ficolin-1 and−3 levels in HIV and HIV/HCV coinfected patients, which indicates a distinct role for these pattern recognition molecules in both viral infections.

## Introduction

Infection by the Human Immunodeficiency Virus (HIV) leads to a chronic disease and, when not treated, to Acquired Immune Deficiency Syndrome (AIDS). HIV/AIDS currently affects 36.9 million people worldwide, with a high rate of mortality and morbidity (1–3). The number of HIV cases has increased despite the decrease in the number of new infection cases, which was estimated to be 3.3 million in 2002 and 2.3 million people in 2012 (1, 2). The implementation of strategies which includes prevention campaigns, increased access to treatment, and the use of less toxic and more efficient anti-retroviral therapies (*highly active antiretroviral therapy*- HAART), have all contributed to increase the survival and decrease the infection rates in risk groups, thereby changing the HIV infection epidemiologic profile in the last decade (1, 2).

Coinfection with other pathogens such as hepatitis B virus (HBV) and hepatitis C virus (HCV), and related diseases are known to be aggravating factors in the clinical condition of HIV patients (4–6). In addition, mortality related to chronic liver diseases has increased significantly in the last decade in HIV patients, with about 24% of AIDS mortality being due to end-stage liver diseases (ESLD), where 66% were attributed to HCV and 17% to HBV coinfection (7–11). It is important to note that these viruses share the same transmission routes, including sexual and blood, both contributing to the high prevalence of HIV/HCV and HIV/HBV coinfection worldwide (6).

The complement system is a key element of innate immunity which plays a crucial role in the host surveillance against pathogens, including HIV and HCV (12–19). Complement comprises a variety of membrane associated and soluble recognition molecules, known as pattern recognition molecules (PRM), and they include Ficolins (Ficolin-1, Ficolin-2, and Ficolin-3), mannose-binding lectin (MBL) and Collectin 11 (CL-K1) (14). These PRMs are able to recognize a variety of pathogen-associated molecular patterns (PAMPs), such as carbohydrates, N-glycan, LPS and sialic acid residues at the microorganism surface as well as endogenous altered cells and double strand RNAs (12, 14), leading to complement activation. This activation may occur through three different pathways: the classic, the alternative and the lectin pathways, which result in a proteolytic cascade that culminates in multiple biological processes including opsonization and phagocytosis of pathogenic agents and altered cells, production of cytokines, inflammation, and induction of adaptive immune response and homeostasis (14–16).

Ficolin-1 is a non-serum type Ficolin that is found as a membrane-associated protein expressed mainly by monocytes and granulocytes, being the less abundant Ficolin in plasma (average of 1.07 μg/mL) (12, 14, 20). On the other hand, Ficolin-3 being the most abundant Ficolin in circulation (average of 26 μg/mL) is a serum type protein expressed in alveolar macrophages type II, bronchial epithelial cells and hepatocytes (12, 21). Several studies showed that Ficolins have an important role in viral infections (17–19, 22–31). Denner et al. (31) reported that incubation of mononuclear cells with HIV-1 immunosuppressive gp41 peptides resulted in low Ficolin-1 mRNA concentrations. The authors suggested that Ficolin-1 may be downregulated by the isu domain of gp41 thereby preventing early local innate response, allowing infection and virus replication (31). A possible role for Ficolin-1 in the protection against HCV infection was proposed in a clinical study by Urban et al. (30), who observed the upregulated expression of Ficolin-1 in chronic HCV patients with *IL28B* rs12979860 CC genotype, which was associated to a favorable response to pegylated interferon-α and ribavirin treatment (30). In addition, Verma et al. (29), demonstrated in an experimental study the binding of Ficolin-3 to influenza A virus which inhibited viral infectivity thereby contributing to host defense against the virus (29). However, there are no prior studies evaluating the role of Ficolin- 1 and -3 in patients with HIV infection, with this now being the first study.

## Materials and methods

### Subjects and samples

This study was approved by the Human Research Ethic Committee of the Clinical Hospital of the Federal University of Parana (1409.074/2007-04). All patients gave written informed consent in accordance with the Declaration of Helsinki. A total of 178 HIV-1 patients (positive for anti-HIV-1, negative for anti-HIV-2 according to Brazilian Ministry of Health guideline) (32) were attending at the Ambulatory of Infectious and Parasitic Diseases at the Clinical Hospital of the Federal University of Parana, in Curitiba, and the Infectology Ambulatory at the Clinical Hospital of the Londrina State University. Among the patients 85 presented coinfection with HCV (Anti-HCV antibody determination by immunoenzimatic micro assay with chemiluminescence QMA Architect—Abbott, USA). As control group, 85 HIV/HCV negative individuals without any clinical complaints were included. The clinical epidemiology data was obtained during the appointments with a questionnaire referring to HIV risk factors and by retrospective analyses of medical records. The following variables were analyzed: age, sex, date of first HIV positive result, possible forms of virus transmission. Same risk factors was also analyzed such as, injected drugs usage, sexual activity, and blood transfusion history as well as clinical progression, T CD4 counts, anti-retroviral treatments and opportunistic diseases (Table 1).

**Table 1:** Clinical and demographic characteristics of controls, HIV and HIV/HCV patients.

**Parameters**	**Patients (*****n*** = **178)**		**Controls (*n* = 85)**
	**HIV (*n* = 93)**	**HIV/HCV (*n* = 85)**	
Median age (years) [min–max]	47 [20–76]	44 [22–71]	38 [18–57]
**SEX**			
Female % (*n*)	43.5 (40/92)	36.9 (31/84)	41.2 (35/85)
Male % (*n*)	56.5 (52/92)	63.1 (53/84)	58.8 (50/85)
**ANCESTRY**			
Euro-Brazilians % (*n*)	81.1 (73/90)	76.2 (64/84)	71.4 (60/85)
Afro-Brazilians % (*n*)	18.9 (17/90)	23.8 (20/84)	28.6 (24/85)
T CD4 cell Counts	193.5 (*n* = 82)	129.5 (*n* = 30)	N/A
HIV Viral load	106500 (*n* = 64)	51100 (*n* = 23)	N/A
AIDS % (*n*)	69.6 (62/89)	75.0 (24/32)	N/A
Progression to AIDS (years) median [min–max]	8.8 [2.7–24]	9.41 [3.0–18.2]	N/A
Opportunistic infection % (*n*)	59.0% (49/83)	61.5% (16/26)	N/A
Bacterial % (*n*)	19.1 (9/49)	12.5 (2/16)	N/A
Viral % (*n*)	8.2 (4/49)	0	N/A
Parasitic/fungal % (*n*)	53.1 (26/49)	66.8 (11/16)	N/A

## Ficolin-1 and ficolin-3 plasma levels

Ficolin-1 plasma concentration was determined by the in-house monoclonal antibody-based method of *time-resolved immunofluorometric assay* (TRIFMA) and were carried out at the Institute of Medical Microbiology and Immunology at University of Aarhus, Denmark (20) on 178 patients (93 HIV and 85 HIV/HCV) and 85 controls. Ficolin-3 plasma levels were determined by the enzyme-linked immune-sorbent (ELISA) assay HK 340 (Hycult Biotechnology, Uden, The Netherlands) in 79 patients (59 HIV and 20 HIV/HCV) and 85 controls. A total of 10 ml of peripheral whole blood was drawn and separated by centrifugation for 10 min at 1,000–2,000 × g in plasma samples using vacutainer plastic blood collection tubes with K2EDTA (BD Vacutainer® Blood Collection Tubes, Curitiba, Brazil).

### Data analyses

Clinical and demographic data was analyzed with GraphPad Prism 3.0 Software (GraphPad Software, Inc., Califórnia, EUA). The distribution of all quantitative variables was evaluated with Kolmogorov-Smirnov and Shapiro-Wilk tests. When normal hypothesis was rejected, medians were compared using non-parametric Mann-Whitney and Kruskal-Wallis and Spearman correlation tests. The associations of Ficolin-1 and -3 levels with HIV and HIV/HCV infection were corrected by multiple logistic regression analysis using STATA 9.2 (StataCorp, Texas, USA). The age, sex, T CD4-cell count and viral load were included as variables in the regression model when the univariate analyses resulted in *p* < 0.2. Ficolin-1 and -3 levels descriptive statistics were presented with medians and percentiles. *P*-values lower than 0.05 were considered statistically significant.

## Results

**Figure 1:**
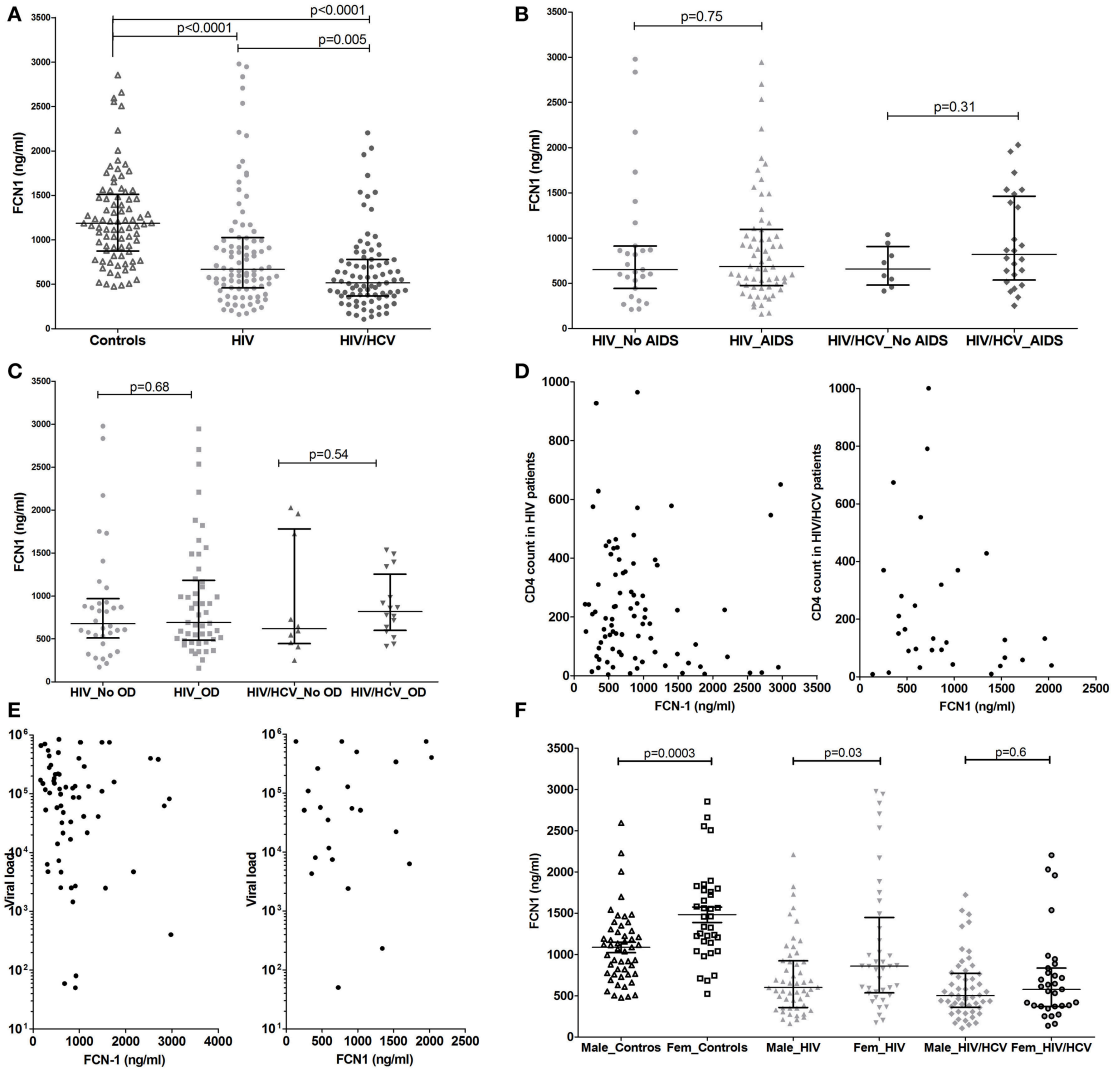
Ficolin-1 plasma levels in controls and HIV infected and HIV/HCV coinfected patients **(A)**, considering the presence of AIDS **(B)** or opportunistic diseases **(C)**. Correlation between Ficolin-1 and T CD4 cell counts **(D)**, Ficolin-1 and HIV viral load **(E)** (Values are in Log10 scale) in patients of HIV and HIV/HCV. Ficolin-1 levels in male and female controls and patients **(F)**.

Ficolin-1 plasma levels were significantly lower in HIV/HCV coinfected when compared to HIV patients (medians of 516 ng/ml vs. 667 ng/ml, respectively; *p* = 0.005) and to controls (medians of 516 ng/ml vs. 1,186 ng/ml, respectively; *p* < 0.0001). HIV infected patients also presented lower levels of Ficolin-1 when compared to controls (medians of 667 ng/ml vs. 1186 ng/ml, respectively; *p* < 0.0001) (Figure 1A). These values were corrected for age and sex with logistic regression and were still significant for the findings above.

The presence of AIDS, opportunistic infection and both T CD4 counts and viral load did not alter Ficolin-1 levels in HIV infected or coinfected patients (Figures 1B–E). There was also no significant correlation between Ficolin-1 levels and AIDS progression time, which was similar for both groups (medians of 8.8 years for HIV and 9.1 years for HIV/HCV patients; *p* = 0.4).

Lower Ficolin-1 levels were observed in males compared to females both in controls (medians of 1,079 vs. 1,457 ng/ml, respectively; *p* = 0.0003) and in HIV patients (medians of 500 vs. 859 ng/ml respectively; *p* = 0.03), but not in HIV/HCV coinfected subjects (*p* = 0.6) (Figure 1F).

**Figure 2:**
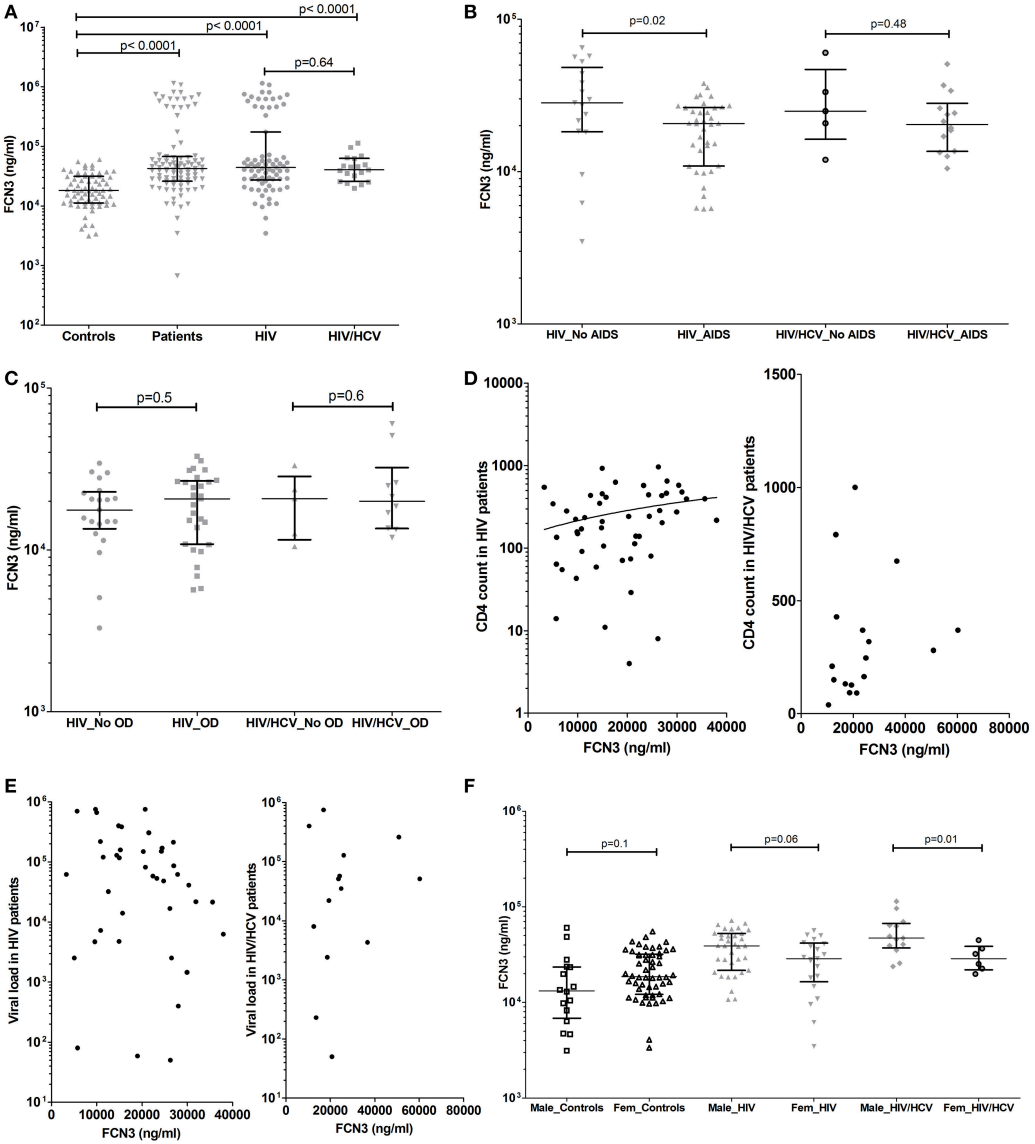
Ficolin-3 plasma levels in controls, HIV patients and HIV/HCV coinfected patients **(A)**, considering the presence of AIDS **(B)** and opportunistic diseases **(C)**. Correlation between Ficolin-3 and T CD4 cell counts **(D)**, Ficolin-1 and HIV viral load **(E)** (Values are in Log10 scale) in HIV and HIV/HCV patients. Ficolin-3 levels in male and female controls and patients **(F)**.

Ficolin-3 levels were significantly increased in HIV patients (medians of 44030 vs. 18240 ng/ml, respectively; *p* < 0.0001) and HIV/HCV coinfected patients (40351 ng/ml; *p* < 0.0001) when compared to controls, with no difference between HIV and HIV/HCV coinfected patients (medians of 44,030 vs. 40,351 ng/ml, respectively; *p* = 0.6) (Figure 2A).

The presence of AIDS was associated with lower Ficolin-3 levels in HIV patients (medians of 20,658 vs. 28,306 ng/ml, respectively; *p* = 0.02) (Figure 2B). A statistically significant correlation was observed between Ficolin-3 levels and CD4 cell counts in HIV patients (*p* = 0.007, *r* = 0.33) (Figure 2D). There was no difference, however, for the presence of opportunistic diseases in HIV or in HIV/HCV patients (Figure 2C), HIV viral load (Figure 2E), and AIDS progression time in both patient groups.

Male HIV/HCV patients presented higher Ficolin-3 plasma levels compared to female (medians of 47,095 vs. 28,693 ng/ml, respectively; *p* = 0.01), with the same trend in HIV (medians of 39,110 vs. 28,784 ng/ml, respectively; *p* = 0.06) but no difference within the control group (*p* = 0.1) (Figure 2F). Ficolin-3 but not Ficolin-1 plasma levels correlated with the age in both groups of patients (*p* = 0.02, *r* = −0.27; HIV *p* = 0.009 *r* = −0.37, e HIV/HCV *p* = 0.65 *r* = −0.04).

## Discussion

We presented here novel evidence that both Ficolin-1 and -3 plasma levels are altered in HIV and HIV/HCV coinfection. Whereas Ficolin-1 levels were found lower in HIV and HIV/HCV coinfected patients, Ficolin-3 were higher in these patients in comparison to controls. Our results indicate that Ficolin-1 and Ficolin-3 may operate as a PRM in a distinct manner facing HIV infection and HIV/HCV coinfection.

These findings are in fact in accordance with the notion that Ficolins—although presenting high structural and molecular homology and specification overlap to PAMPs binding—exhibit differences in functional activities and in the pattern of tissue expression, with consequences in the potential to activate complement as well having other roles in the immune response (14). Ficolins can interact with viral glycoproteins which are constituted of N-Acetylglucosamine (GlcNAc), such as gp120 and gp41, which are essential for cell binding and infection onset, but is also a target for Ficolin-1 and Ficolin-3 (17–19). Considering that PRMs bind to these viral surface glycoproteins, they may have a role in the process of HIV infection. In fact, complement may play different activities in HIV pathogenesis, such as blocking virus entrance into the cell, signalizing phagocytosis, inflammation, as well as forming the lytic complex at the infected cell membranes (17–19, 22–31). On the other hand, viruses may bind to complement proteins impairing an effective adaptive immune response thereby facilitating its entrance into target cells through complement receptors (CR) (17–19, 22).

Studies relating the role of Ficolin-1 and -3 in viral infections are scarce (29–31) and to our knowledge, this is the first study evaluating the levels of Ficolin-1 and -3 in HIV and HIV/HCV coinfected patients. The low Ficolin-1 levels observed in HIV and HIV/HCV patients suggests possible protein consumption due to viral infection. It is known that neutrophil autocrine Ficolin-1 can bind to CD43 (a neutrophil membrane sialoprotein) inducing neutrophil adhesion at the beginning of an inflammatory response (14, 33). On the other hand, in the late phase Ficolin-1 was shown to have strong affinity to C-reactive protein resulting in downregulation of pro-inflammatory cytokines (34, 35). In addition, Ficolin-1 levels could be reduced due to downregulation effect of the viruses as described for HIV gp41 immunosuppressive (isu) domain (31). Interestingly, Ficolin-1 levels were even lower in HIV/HCV patients when compared to HIV. It is known that HCV coinfection is an aggravating factor in the clinical condition of HIV patient exacerbating the existing inflammatory process (4–6), and that complement can interact with glycoproteins of both virus (17, 19, 25). Thus, our results suggest that low Ficolin-1 levels might be a consequence of the interaction with both HCV and HIV in addition to an immunomodulatory effect due to chronic inflammatory process.

Meanwhile, the elevated Ficolin-3 levels found in HIV and HIV/HCV patients compared to controls may derive from compensatory mechanisms of upregulation of this protein due to its interaction with viral glycoproteins as well as complement activation. Ficolin-3 binding to HIV or HCV has not yet been reported, however, it is known that HIV gp120 is rich in fucose, which is a particular ligand of Ficolin-3 (36). Still, high expression of Ficolin-3 found in both viral infections could be related to the inflammatory status seen in co-infected patients, contributing to the chronic process of these conditions. Elevated Ficolin-3 levels were also observed in ovarian tumor patients (37), related to shorter graft survival after kidney transplantation (38), in patients with Leprosy (39) and Systemic Lupus Erythematosus (40, 41), all conditions associated with inflammatory process.

Higher Ficolin-1 levels in female HIV patients and controls corroborates previous findings (42), but, such difference was not observed in HCV coinfected patients, what could be due to the higher male frequency in this group. Ficolin-3 levels were higher in males only in the co-infected group, a sex difference previously observed in healthy subjects (42).

This study has some limitations. First, polymorphisms shown association to Ficolin-1 (rs7857015, rs10120023, rs10117466) (43, 44) and Ficolin-3 (rs532781899, rs28362807, and rs4494157) (39) concentrations, that could have contributed to the variation of protein concentration in HIV and HIV/HCV patients, were not assessed. Future studies in this cohort of patients should include the investigation of these variants. Second, although Ficolins concentration showed significant results among the groups the relevance of these findings should be confirmed in a larger sample of patients, and, in experimental studies showing Ficolins 1 and 3 interaction with both HIV and HCV. Third, the status of HCV co-infection was based only on anti-HCV test, which is an evidence for prior exposure to HCV. Since all patients were attending the HIV ambulatory whose routine for HCV is based on serology, molecular tests were not available at the time of the study. In order to established direct evidence of current infection both the presence of HCV RNA and a longitudinal follow-up in HIV/HCV patients should be considered.

Nevertheless, our data represent a pioneer study on the role of Ficolin-1 and -3 in HIV/HCV infections. This novel finding suggests that these proteins contribute in a different manner to host defense against these viruses and may be helpful for future studies in understanding the function of Ficolins in HIV/HCV infection as well as in the development of new therapeutic targets.

## Author contributions

IM-R, ST, JJ, and MT contributed with conception and design of the study. MT, HM, MB, and FA executed laboratory procedures. KL performed the statistical analysis. MT, KL, FA, and MB wrote the original draft of the manuscript. ER performed the recruitment of patients. All authors contributed to manuscript revision, read, and approved the submitted version.

### Conflict of interest statement

The authors declare that the research was conducted in the absence of any commercial or financial relationships that could be construed as a potential conflict of interest.
